# Primary care physicians’ perspectives on digital health tools for chronic disease management: A rapid review

**DOI:** 10.1371/journal.pdig.0001085

**Published:** 2025-11-20

**Authors:** Derya Demirci, Muhammad H. Minhas, Cynthia Lokker, Catherine Demers

**Affiliations:** 1 Faculty of Health Sciences, McMaster University, Hamilton, Ontario, Canada; 2 Schulich School of Medicine & Dentistry, University of Western Ontario, London, Ontario, Canada,; 3 Department of Health Research Methods, Evidence, and Impact (HEI), McMaster University, Hamilton, Ontario, Canada; 4 Division of Cardiology, Department of Medicine, McMaster University, Hamilton, Ontario, Canada; Iran University of Medical Sciences, IRAN, ISLAMIC REPUBLIC OF

## Abstract

Chronic disease management is a burden for many patients. Digital health tools (DHTs) can leverage technology to rapidly develop and disseminate interventions to alleviate obstacles faced and promote self-care. Primary care physicians (PCPs) are most directly involved in the care of chronic disease patients; however, their perspective is often overlooked. To develop an effective DHT for chronic disease management, PCP attitudes are critical to ensure improved patient integration, adoption and care outcomes. The purpose of this rapid review is to explore and identify PCPs’ perspectives and attitudes regarding DHTs for chronic disease management and generate major themes from our findings using key literature. The themes will be used to guide DHT creators, clinicians and policy makers on adoption and implementation considerations. We conducted a rapid review of primary qualitative research between 2000 and 2022. Two reviewers, independently, conducted study screening, selection, and data abstraction. The themes identified in the articles were extracted and presented narratively. The data was analyzed using NVIVO12 software. Braun and Clarke’s deductive thematic analysis was used, and the themes identified were extracted and presented narratively. Nine qualitative research studies met the inclusion criteria. Themes were classified into two major categories: physician–patient relationship and physician–technology relationship. Within these, seven subcategories were identified: (1) Increased Physician Workload, (2) Data Capture & Data Quality, (3) Evidence-Based Care, (4) Education and Training, (5) Liability, (6) Patient Interactions, and (7) Patient Empowerment and Suitability. DHT creators/endorsers need to consider how DHTs affect the patient–physician relationship and the physician–technology relationship as this affects how PCPs perceive DHTs. PCPs’ perspectives must be taken into consideration to promote self-care for patients living with chronic diseases.

## Author summary

Our team searched current literature to understand primary care physicians’ needs and attitudes toward health technology, more specifically digital health tools, created for patients living with chronic diseases. It is important to understand primary care physician attitudes as they are in frequent contact with their patients with chronic diseases. In fact, patients with chronic diseases who visit their primary care physician often can help reduce hospital admission rates. The findings from this rapid review will be crucial for a better understanding for considerations regarding digital health solutions for patients with chronic diseases. It will shed light on what primary care physicians need to do to support their patients, and what tool makers need to consider when creating and implementing digital health tools for patients trying to manage their condition. Our major insights and findings must be integrated into digital health tools/platforms that promote self-care, in order for them to be successful and adopted by key stakeholders such as primary care physicians and organizations.

## Introduction

### Background

Digital health is the use of information and communication technology in medicine to manage illness and promote wellness [1]. It is a multidisciplinary field that continues to evolve with technological advances and growing data availability [2]. Digital health tools (DHTs) enable healthcare providers an avenue to provide care beyond traditional methods. The COVID-19 pandemic was a major catalyst for change in digital health, driven by restrictions and strain on the healthcare system [3]. These consequences constrained access to care, thereby accelerating the adoption of digital technologies and influencing the culture of healthcare delivery for both providers and patients [3]. The pandemic ultimately brought unique opportunities and was described as the first global pandemic of the digital age [4]. Within a short period, the pandemic brought about years of advances through DHTs [4]. However, adoption was not equal across regions, as differences in infrastructure and resources shaped how quickly DHTs could be integrated into care [4]. Importantly, these advances extended beyond virtual care, with DHTs increasingly used to support chronic disease management through innovations such as remote monitoring, digital self-management platforms, and hybrid care models, trends that have continued post-pandemic [5].

DHTs promote self-care in patients living with chronic disease. Patient engagement has been shown to improve health outcomes in patients, especially those with chronic diseases [6]. Chronic diseases describe conditions that last for a year and/or more in which patients require ongoing medical care [7]. Self-care, which embraces self-maintenance, self-management, and self-confidence, involves behaviors that maintain physiological stability and respond to symptoms when they occur [6]. Often, patients with chronic diseases such as diabetes mellitus and heart failure (HF) require self-care practices which can be accomplished through DHTs. In Canada, HF is associated with a significant economic burden, accounting for up to 2% of annual healthcare expenditures ($2.8 billion/year) [8], with hospital admissions representing approximately 70% of the cost of treatment [9]. As DHT tools empower patients to actively manage their health, they have the potential to improve their quality of life, reduce hospital readmissions, and related healthcare costs. A systematic review of 54 studies found that digital health interventions have improved health behavior, clinical assessment, treatment compliances and enhanced coordination of care [10].

Primary care plays a central role in patient education and behavior change, as individuals with chronic diseases most often engage with the healthcare system through this setting. Primary care is defined as integrated and accessible healthcare services by clinicians and is often the foundation and the first point of contact for patient needs [11]. Hence, it is crucial to investigate and consider primary care physicians’ (PCPs) perspectives when developing and implementing DHT tools for patients with chronic diseases. Unfortunately, this is a gap in research as many studies fail to report the perspectives of various key stakeholders within health care, including PCPs [12]. Ultimately, a key factor in ensuring the successful adoption of DHT for patients involves understanding clinicians’ perspectives to support and educate them on DHT tools [13].

The purpose of this rapid review is to examine PCPs’ perspectives on digital health solutions for chronic disease management, synthesize major themes from the literature, and provide recommendations to inform PCPs and DHT developers. Although previous review studies have investigated DHTs for chronic diseases [14,15], there has been no comprehensive review of PCPs’ perspectives and attitudes toward DHTs in the context of chronic disease. Moreover, for the purposes of this review, DHTs were defined as technologies that support the management of chronic health conditions and involve PCPs in their use, oversight, or integration into clinical workflows.

## Methods

A rapid review was undertaken to identify and analyze PCPs’ perspectives on DHTs and interventions that support patients with chronic diseases to promote self-management. This rapid review was guided by the Cochrane Rapid Review Methods Group [16,17]. Rapid reviews provide information in a timely manner compared to systematic reviews as they use systematic search strategies but limit particular aspects of the systematic review process, such as the breadth of databases searched, the number of reviewers involved, and the inclusion of gray literature [15] . We conducted a rapid review because our research question is specific, lacks extensive literature and is primarily narrative-based. Additionally, we omitted formal quality assessment of studies. Given the rate of innovation of technology and the rapidly changing nature of DHTs, we felt this methodology was the most appropriate for timely dissemination in a swift-moving research area. We screened articles using our inclusion criteria and undertook a thematic analysis of healthcare provider perspectives to synthesize and report on the current literature. The results were then collated, summarized, and reported. The eligibility criteria, searching, study selection, data extraction.

### Eligibility criteria

We followed the PICO Framework in establishing eligibility criteria (Table 1). We considered any primary qualitative research peer-reviewed articles that included PCPs’ perspectives on digital health interventions aiming to support patients with chronic diseases, published in 2000 or after. The language was restricted to English, and study locations were limited to the USA, Europe, Australia, or Canada to ensure the relevance and comparability of the studies, as these regions share similar healthcare frameworks and digital health trends.

**Table 1:** PICO eligibility criteria.

Population (P)	Primary Care Physicians
**Intervention (I)**	Digital health tools for chronic disease management (e.g., diabetes, ischemic heart diseases, cerebrovascular diseases, chronic, obstructive pulmonary disease, asthma, hypertension, dyslipidemia, arthritis/ rheumatoid arthritis, chronic pain, cancer, chronic renal disease, inflammatory bowel diseases, mood disorders and attention deficit disorders). We will rely on the authors’ definition of chronic disease and presenting, or at risk of presenting, a concomitant mental health problem (e.g., mood disorders, depression, anxiety, obsessive-compulsive disorder, panic disorder and post-traumatic stress disorder).
**Comparator (C)**	No intervention
**Outcomes (O)**	Thematic analysis of PCPs’ perspectives and best practices to support PCPs in managing patients with chronic diseases utilizing DHTs

### Literature search strategy and terms

Our team initially searched two databases, including Medline—Ovid and Pubmed and Scopus. We first brainstormed relevant keywords to use in our search which were: “Digital Health,” “Family Physicians,” “chronic diseases management,” “perspectives,” and “self-management.” We then replaced these terms with controlled vocabulary according to Medical Subject Heading (MeSH) shown in Table 2. We decided to adopt the search terms “Telemedicine,” “Physicians, Primary Care,” “Chronic Disease,” “Attitudes,” “Self-Management,” and “General Practitioners” to increase our indexing results within the databases. The term ‘PCPs perspective’ is used throughout the review, reflecting feedback from individuals using eHealth technologies for the provision of care. For example, the opinions or experiences of providers may be included. To supplement our literature search, we used our search terms within google scholar to increase our reach of research articles. The terms “eHealth,” “telehealth,” “telemedicine,” and “digital health” are often used interchangeably, with all terms generally referring to the electronic means of receiving or giving care, such as the use of video conferences or digital imaging technology [18].

**Table 2:** Key search terms for literature search.

Term	MeSH Term
Ehealth, digital health	Telemedicine
Primary Care Physicians	Physicians, Primary Care
Chronic Disease Management	Chronic Disease
Perspectives	Attitude
Self-Management	Self-Management
General Practitioners	General Practitioners

### Screening

**Fig 1:**
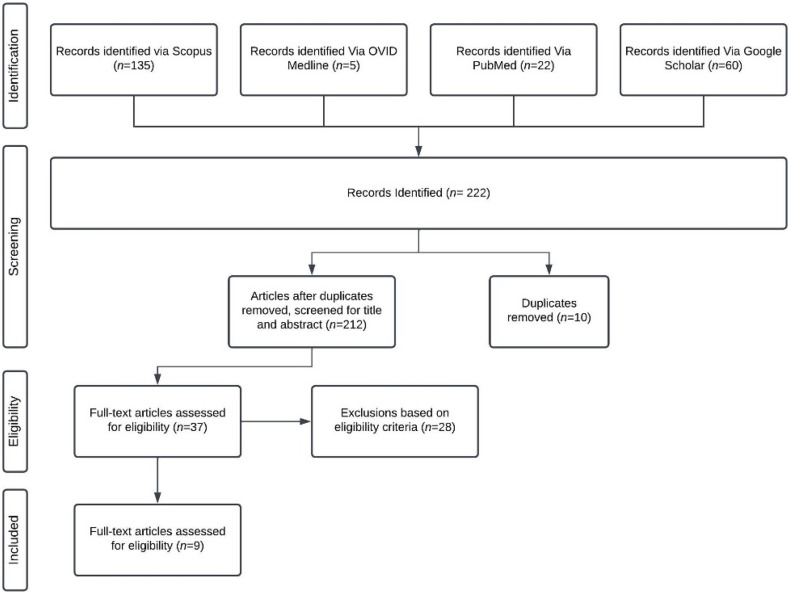
Disposition of articles and literature screening flow diagram. This flow diagram depicts the process and flow of information (including number of records of identified, included, and excluded study numbers) through the phases of the rapid review.

The screening process was conducted by **2 reviewers** independently of each other. Both reviewers identified the same set of eligible studies at each stage: 37 articles in the initial screening and 9 after full-text review. Each article was initially screened according to the title and abstract cross-referenced to our inclusion criteria. The article must have included perspectives of primary care and/or family physicians, chronic diseases, and DHTs to be eligible for further screening. Each article that met this criterion (*N* = 37) was then inputted into a standardized extraction form for full-text screening. After a full-text review by both reviewers independently, the resulting identified studies (*N* = 9) were analyzed and extracted for major themes. Two reviewers independently conducted coding and identified key themes. Themes were identified and further refined through a collaborative process between both reviewers and verified by a third party. The focus of the papers must have included a discussion about DHTs in relation to PCPs managing patients with chronic diseases; themes had to be relevant to PCPs’ perspectives and needs. Any discrepancies were resolved through discussion involving a third party. Fig 1 displays a PRISMA chart to outline the rapid review process which is an adapted PRISMA flow diagram of the study inclusion process. Please refer to the appendix.

### Data abstraction

To organize and chart the data, data abstraction was conducted in duplicate by two reviewers. Studies were classified based on relevant criteria, including the type of intervention, and type of study. The first-person perspectives of providers were then collated, and prevalent themes were identified from the article text and included direct quotations. These themes were identified by two independent reviewers and through a collaborative process, the themes were refined and reorganized. The major categories identified were physician–technology relationships and physician–patient relationships. Each theme was further divided into subcategories based on similar thematic perspectives from PCPs.

## Results

We identified nine articles that met our inclusion criteria. Based on the literature reviewed, ***7 major themes*** were identified. These themes were further subcategorized as ***Physician–Technology relationship*** or ***Physician–Patient relationship*** category. The major themes identified were physician workload, data capturing and quality, evidence-based care, education and training, liability, patient interactions, and lastly, patient empowerment & suitability.

Table 3 outlines each article with the main themes identified.

**Table 3:** Study characteristics of the papers included.

Authors (year)	Country	Sample size	Main themes
Ayre et al (2019)	Australia	*n* = 25	**Increased physician workload:** • PCPs believe that workload will increase as they need to learn to use the app and provide care remotely • Concerns about lack of incentive if doing extra work • Workflow integration as crucial **Liability concerns** • Monitoring of data outside work hours Patient empowerment and suitability • Allow patients to take control and manage their health • Suitable for younger, more independent patients **Data capturing and quality** • Provide summary of data • Enhanced documentation of patient data **Patient–physician relationships** • Concerns that the too would undermine patient–physician relationship • Enhanced communication and stronger relationship **Education and training** • Allow for key messages during consults to be retained by patients • Enhancing education for participants regarding their health as beneficial
Barber et al (2019)	Canada	*n* = 4	**Increased physician workload** • Educating patients on application function, setting up Wi-Fi for clinic **Patient empowerment and suitability** • Concerns about causing anxiety of older patients **Liability** • Accountability capabilities for patients
Bhattarai et al (2020)	Australia	*n* = 17	**Data quality** • Might lead to data overload, physicians prefer data summaries beneficial • Physicians prefer a range of data points **Evidence-based care** • Physicians require evidence to prove tool effectiveness and a regulatory body **Education and training** • Physicians want to be familiar with the tool **Patient empowerment and suitability** • Concerns about age, technology literacy for patients using DHTs • Empowered patients
Morrissey et al (2018)	Ireland	*n* = 10	**Increased physician workload** • Expanding duty of care for overworked physician through DHT **Data capturing** • DHT provide accurate blood pressure readings • Easier to convey information through visualization of data **Patient empowerment and suitability** • Suitable for patients who already tech-savvy, independent • Motivated patients to manage their health **Education and training** • PCPs to know about the tool before recommending it **Patient–physician relationship** • Power dynamic being disrupted **Liability** • Concerns about monitoring data after hours
Slevin et al (2020)	Ireland	*n* = 32	**Evidence-based care** • Regulatory guidelines needed for DHT **Data quality** • Concerns about lack of validation and calibration • Data reliability concerns **Workload** • Resource constraints **Patient empowerment and suitability** • Digital literacy concerns
Van de Vijver (2022)	Netherlands	*n* = 4	**Patient empowerment and suitability** • Not suitable for patients with hypochondria and those with low digital literacy • Patients having an active role in managing their health **Workload** • Increase in work efficiency as the traditional model was seen no longer feasible • Also, would increase workload due to administrative works, messaging features, etc.
Varsi et al., (2021)	Norway	*n* = 12	**Patient empowerment and suitability** • Barriers to usage: cognitive impairments, technological illiteracy, advanced age, cultural barriers, linguistic barriers, equipment that not everyone can afford
Grant et al., (2019)	United Kingdom	*n* = 11	**Increase workload** **Liability and Security** • Patient confidentiality, especially when using SMS texting **Patient–physician interaction** • Enhanced communication between patients and HCPS • Enhanced doctor–patient relationship **Data capturing and data quality** • Useful clinical data that allows for more intense follow-ups • Easy access to patient data
Duineveld et al (2016)	Netherlands	*n* = 20	**Patient empowerment and suitability** • Not all patients wanted to be more in charge, for those young and educated • Patient empowerment • Relief for patients to be educated on their symptoms **Patient–physician interaction** • The tool as a supplement to medical care and not as a replacement **Increase workload** **Education and training** • Be knowledge on the tools content **Data capturing and data quality** • Some expressed wanting to see results intensively and receive alerts when the status is critically low and some acted more as a consultant

### Physician–technology relationship

#### Physician workload.

Our review reveals that many PCPs within our selected studies included in this review expressed concerns about increased workloads as a result of supporting the adoption of DHTs for themselves or their patients [19–25]. With the emergence of the COVID-19 pandemic, many HCPs’ workloads, especially PCPs have increased significantly which poses an obstacle in adopting complicated and time-consuming digital patient tools [26]. PCPs described the management of data and logging into separate web portals as time-consuming [24]. Many PCPs specifically expressed concerns of being responsible for promoting tools, providing patient education/training, investing additional resources within their clinic, monitoring patients/tools outside of work hours, and integrating data within their clinic Electronic Medical Record (EMR) system. PCPs perceive these resources and workload concerns as barriers to adopting DHTs to support their patients. PCPs noted that integrating mobile applications for older adults can be time-consuming, as they often felt the need to download and familiarize themselves with the app before recommending it to patients [27]. Adoption of DHTs is perceived by PCPs to encourage expanding duty of care through longer working hours and increased communication/monitoring of patients. PCPs also felt the need to invest in resources such as Wi-Fi capabilities for their clinics as a necessity [20]. Lastly, PCPs stated a need for having patient data collected by DHTs to integrated into their existing EMR systems as manual integration would increase workload for the care provider and clinic [19].

#### Data capturing and data quality.

DHTs can capture large amounts of data that may be valuable for PCPs. DHTs allow patients to collect beneficial data at home to help PCPs make clinical decisions and empower patients to keep records of their own health [19,21–25,27]. This in turn could allow for more productive follow-ups as there is easier access to clinical information [24]. Often, PCPs face challenges when obtaining clinical data such as blood pressure readings as they can be inaccurate in the clinic versus at the patient’s home. However, allowing PCPs to have access to clinical data across different time spans was deemed helpful [21]. Although there were many benefits to real-time data sharing, this might lead to data overload for PCP [27]. As a result, PCPs preferred a summary of key patient data during consults to make clinical decisions as opposed to large reports, which can be burdensome to read and interpret [27]. Some concerns regarding data from DHTs involve lack of calibration, validation, and reliability which may lead to patient safety concerns. For instance, if patient data demonstrate inaccurate results, then this may introduce health anxiety for patients [22]. Reliability concerns were in combination with privacy concerns when DHTs incorporated SMS messaging features [24–28]. Overall, some PCPs perceived DHT data to be beneficial in guiding PCP decision-making, if presented in manageable chunks, and not overwhelming, while other PCPs questioned the quality and validity of data from DHTs.

#### Evidence-based care.

Within our search, it is evident that PCPs expect DHTs to be high-quality and evidence based**.** There are many DHTs available, but unfortunately not all provide the background research to support their claims. Evidence-based care is defined as integrating research-based evidence in the decision-making processes and PCPs want to see significant results that demonstrate a positive impact on patient health [27]. Moreover, PCPs require evidence-based DHTs or applications to provide their endorsement of the tool to their patients [27]. Supplemented with evidence-based care, an authority figure or regulatory body will also increase the likelihood that PCPs will adopt and promote the utilization of DHTs for patients in their care [22].

#### Education and training.

Education and training are a major theme observed across many of the studies reviewed. PCPs expressed a need to be educated and trained on the DHTs that their patients are utilizing. PCPs would ideally like to familiarize themselves with DHTs and receive basic education or training before promoting it to their patients [21,25,27]. Moreover, digital tools can be informative for patients and are a valuable resource for older patients to promote education and for PCPs to facilitate information and instruction regarding health [27]. Although PCPs favored face-to-face care over online care [19], the supplementation of a mobile application during consultations was viewed positively. PCPs were interested in a mobile application to provide patients with brief educational materials that included key messages that were discussed during consultations [19].

#### Liability.

Although many PCPs expressed the positive value DHTs can have for their patients, there were strong concerns about liability. Specifically, many physicians expressed liability concerns regarding the recommendation of DHTs [23] and the responsibility of monitoring/receiving data after work hours [21,25,27]. PCPs expressed that they felt liable if they were to recommend a DHT to patients and if they were to view the data from these tools as part of their EMR systems [25]. PCPs also felt that they would need to have enough information on the app/tool before recommending it to their patients [22]. There is also the concern that if PCPs receive alerts of patients’ data, they will feel a sense of responsibility to act and respond if patient symptoms appear to be deteriorating [22], and as a result, they may be held liable. Moreover, accountability capabilities for patients would be deemed attractive as it would shift the responsibility away solely from the physician and more to the patient [28]. Lastly, a confidentiality risk exists when using DHTs with SMS texting or messaging to communicate with patients [24].

### Physician–patient relationship

#### Patient interactions.

PCPs expressed concerns about dehumanization and power dynamics being disrupted [19,21]. Moreover, patients may rely on possible incorrect information from DHTs and disregard their physician’s professional opinion. PCPs have emphasized that DHT should supplement medical care and not replace it [25]. Alternatively, DHTs can also positively impact patient–physician relationships as they make difficult conversations regarding their health easier [21,24]. Having a tool also helped focus the conversation on patient goals, enhancing communication which promotes a positive patient–physician relationship [19,24]. Digital health solutions were seen positively in this aspect.

#### Patient empowerment, suitability, and factors.

Many PCPs believe that DHTs can empower patients to manage their chronic illnesses. PCPs believe that having access to visual health information will motivate patients to change their lifestyles and adhere to their medication [21,25]. However, PCPs also believe DHTs are not readily adoptable by all patients. Some studies discuss that PCPs feel giving patients access to health data or too much information might result in unnecessary anxiety or concern [21,29]. Other studies have mentioned that patients able to adopt these tools are younger, technologically savvy, and already highly motivated about their health [21,25,29]. PCPs expressed patient factors such as age and technology literacy are important in determining suitability for DHTs. They expressed reluctance to recommend it to older populations as they question older individuals’ technological ability and the impact these tools may have on individuals who need it the most [19,21,23,25]. However, PCPs also described the positive impact DHTs have on empowering patients and motivating them to manage their health.

## Discussion

This rapid review identified a variety of themes associated with PCPs’ perspectives and attitudes on DHTs for chronic disease management. DHTs designed for patients should ultimately aim to improve the quality of care and health outcomes. Our results demonstrated a key theme discussed widely in literature, physician workload. One major barrier to DHT adoption for physicians is workload concerns expressed by HCPs as it may disrupt current processes [30]. A survey conducted in 10 countries found that the pandemic increased PCPs’ workload, patient backlog, and administrative duties [31].This impacts the quality of care they can provide to patients, with findings demonstrating that PCPs experiencing burnout symptoms were significantly more likely to state that their quality of care has decreased [31]. Critical ways to reduce burnout are the following: provide training, reduce documentation & task time, expand care time and implement quality improvement initiatives to allow time for DHT adoption [32]. Additionally, the lack of physician reimbursement is also a barrier to adoption. However, this can be reduced by creating incentive structures to include supporting patients with DHTs [33]. PCPs should be acknowledged and incentivized for their work through fair compensation. Therefore, DHTs should be designed to mitigate PCP workload or incorporate mechanisms for appropriate reimbursement, acknowledging the existing burden on physicians. Creating a DHT that is simple and can easily integrate into current systems utilized by PCPs will help ensure appropriate workload.

Another crucial aspect of an effective DHT is **data capturing and data quality.** Primary care consists of four functions: comprehensiveness, first contact, continuous longitudinal relationship, and coordinated care [34]. PCPs found patient data valuable, as it provided a holistic view of clinical information, supported clinical decision-making, and facilitated comprehensive, longitudinal patient care [21]. This provides PCPs with important clinical data for enhanced decision-making. However, DHTs can present data in ways that can be overbearing, therefore, PCPs prefer patient data to be reported in summaries and manageable chunks to help guide their consultations with patients [27]. Generally, the data obtained in primary care is crucial and a key enabler in improving the data captured is to build trust between key stakeholders such as patients, clinicians and third-party data end-users. Factors such as stakeholder engagement, transparency, strong data security and protection should be taken into consideration to ease PCP’s concerns [35]. Ultimately, the data obtained from DHTs are crucial, but PCPs’ concerns need to be reduced to provide them with adequate support in managing patients.

Moreover, PCPs expressed the importance of **evidence-based care**. PCPs expressed the need for validation through research and scientific trials that would confirm the effectiveness of the tool and outline the positive impact it would have on their patients. Credibility and evidence-based tools backed by high-quality research studies are deemed essential as this would likely allow PCPs to trust the tool, therefore encouraging the adoption and promotion of DHTs by PCPs towards their patients [23]. Evidence-based tools can enhance PCPs’ trust by alleviating concerns about liability and reducing the burden of demonstrating effectiveness, as their use is supported by research-backed claims. This underscores the need for scientific studies to validate the tool’s effectiveness. Evidently, DHTs need to provide evidence-based care that consists of the most current guidelines and quality measures for PCPs to confidently endorse them [36].

In relation to **liability**, PCPs expressed liability concerns in both, initially recommending a tool to their patients and in monitoring patient data [19–21,24]. Ensuring clinical guidelines are in place and adhered to, would ensure that there are set expectations between all parties involved, reducing any liability concerns. Ideally, patients/tool creators would take accountability for patient safety and data accuracy as this would help reduce PCPs’ concerns of being expected to monitor patients’ data after hours, reducing liability and workload which would allow PCPs to more comfortably endorse the DHT [20]. Another possibility would be to have patients sign consent forms that would enable PCPs to disclaim liability for after-hours data monitoring [37]. Nonetheless, DHTs need to be mindful of ensuring there are set guidelines and expectations in place for all parties involved as PCPs are not comfortable accepting additional liability [38].

**Education and training** are also critical as there are many DHTs available in the market, making it challenging for PCPs to remain up to date on every tool available that their patient may want to use. Ideally, a digital tool should educate PCPs about the capability and features in a simple, convenient manner. Moreover, PCPs should be educated on the risks and challenges such as increased workload, data sharing, and confidentiality. Teaching material for DHTs should be reviewed and remain current with technology development [39]. Education and training could take the form of a short video, webinars, or a one-page pamphlet that contains the necessary information for PCPs [40]. Additionally, DHTs can also educate patients about their conditions through incorporating educational materials within their devices. This would help PCPs ensure that their patients remain educated and knowledgeable about their condition [19,25,27]. Moreover, a platform that includes PCP’s ability to facilitate disease management information could be seen as positive if other key factors such as physician workload and liability issues are addressed.

**Patient interactions** were also a critical theme based on our review. PCPs expressed concerns regarding the relationship being disrupted as patients may provide more importance on the information of the DHT as opposed to their PCPs. This needs to be taken with caution because DHTs should be seen as supplementary to clinical care. Some recommendations include setting expectations that DHT’s role is to help support patients, and ultimately PCPs should be responsible for clinical interventions [41]. Positively, many PCPs expressed the impact it had on communication, making difficult conversations easier and education in DHTs helpful for both patients and PCPs [21]. DHT creators should ensure that patients are educated and empowered, yet still ensure that there is a balance between compliance and autonomy [41]. PCPs should not feel undermined by the DHTs, and emphasis should be on the PCPs recommendations/health advice, and DHTs should be expressed as supplemental tools to care.

Lastly, regarding **patient empowerment and suitability,** our review illustrated that many physicians believe that patient factors are important in determining digital health suitability, especially for older patients. PCPs have expressed that older populations are limited in DHTs usability due to technological literacy and capabilities restraints. However, older patients have expressed positive attitudes toward DHTs, and the impact tools could have on their health [40]. Yet according to our review, PCPs often make assumptions about what their patient can or cannot do. PCPs have expressed that they did not want to worry and/or challenge older patients with DHTs as they did not want to heighten their anxiety [20,22]. Although barriers such as digital literacy, and physical and cognitive deficits do exist, DHTs need to consider these when catering to the older population. PCPs should also make fewer assumptions about their patients’ capabilities because if provided with the opportunity to increase their well-being, patients are accepting of utilizing DHT to do so [42]. Overall, DHTs should consider diverse patient needs as PCPs become hesitant to recommend a technology tool for specific patient populations. PCPs should be mindful and ensure that they make fewer assumptions about their patient’s capabilities.

With chronic disease management, DHTs promoting self-care should consider each of the themes that were deemed important when implementing a self-care DHT. This would not only benefit both the PCPs and the patient but also further promote the adoption and utilization of digital self-care tools which can truly make meaningful differences.

## Limitations

The limitation of our study was that because our research question was narrow, this limited the number of articles that met all our inclusion criteria. As a result, our findings were primarily based on a small number of articles (*N* = 9) and may be difficult to extrapolate the themes. In addition to a small study size, many of the studies had a small number of healthcare professionals as participants, as well as a heterogeneous mix of DHTs such as mobile applications, tools and interventions. This might provoke different responses and attitudes in different clinicians. Another limitation is that, due to the nature of the rapid review and in line with Cochrane guidelines, a formal quality appraisal and detailed comparison of study designs were not conducted, which may affect interpretability and comparability across studies.

### Next steps

Future research should explore the impact of DHTs not only focusing on physicians but also on other allied healthcare professionals, such as nurses. This would facilitate an investigation into the diverse range of DHTs available to improve patient outcomes, particularly those that do not directly involve PCPs. Additionally, it is crucial to examine how marginalized groups are affected by the implementation of DHTs, ensuring equitable access and outcomes for all patient populations. Future reviews could also benefit from incorporating bias appraisal, for example by using streamlined risk-of-bias tools or concise critical appraisal checklists, even within rapid methods, to strengthen the reliability of findings.

## Conclusion

To the best of our knowledge, our study is the first rapid review that explored the PCPs’ perspectives on DHTs for patients with chronic diseases through literature reviews. Findings revealed the impact on physician workload as a key factor in whether PCPs view the DHT positively or negatively. There are many aspects regarding the data that DHTs create such as its reliability and providing PCPs with only important clinical information. Digital tools should be supplemented with evidence-based, have clear clinical guidelines in place and provide education and training for PCPs. PCPs often consider patient digital literacy and the impact the DHT would have on their patient–physician relationship; ensuring that they advocate a tool that would be helpful for their patients while maintaining a positive patient–physician relationship. Future DHTs must consider the themes discussed within this review to successfully be supported and endorsed by PCPs who are the primary support for patients with chronic diseases.
